# Intraoperative Verbal Communication in Pediatric Single‐Incision Laparoscopic Percutaneous Extraperitoneal Closure: A Comprehensive Analysis and Educational Implications

**DOI:** 10.1111/ases.70170

**Published:** 2025-10-27

**Authors:** Masanaga Matsumoto, Yohei Sanmoto, Kouji Masumoto

**Affiliations:** ^1^ Department of Pediatric Surgery University of Tsukuba Hospital Tsukuba Japan; ^2^ Graduate School of Comprehensive Human Sciences University of Tsukuba Tsukuba Japan

**Keywords:** education, laparoscopy, pediatrics

## Abstract

**Introduction:**

In pediatric surgery, declining case volumes and restrictions on working hours have intensified the need for efficient training strategies. The operating room remains a central educational environment, yet the nature of intraoperative teaching is unclear. We examined the educational role of intraoperative verbal communication and identified opportunities for improvement.

**Methods:**

We retrospectively analyzed data of unilateral single‐incision laparoscopic percutaneous extraperitoneal closure procedures performed between December 2024 and June 2025 with complete audio and video recordings. Verbal statements were transcribed verbatim, classified by type and content, and analyzed for overall distribution, phase‐specific frequency, composition of attending‐to‐operating surgeon communication, and intraoperative debriefing occurrence during the wound closure.

**Results:**

Nineteen cases were included, yielding 7374 statements. The most common content category was General (39.6%), followed by Instrument handling (19.1%), Anatomy (14.9%), and Operation method (14.8%). The proportion for Instrument handling increased to 27.1% during laparoscopic manipulation; that for Private talk rose to 18.5% during wound closure. Communication from attending to operating surgeons was most frequent in the laparoscopic manipulation phase (median, 3.4; interquartile range, 2.6–4.7, per minute), with higher proportions of Commanding (11.8%) and Advising (14.9%) statements. Intraoperative debriefing on the preceding laparoscopic phase occurred in only two cases (10.5%).

**Conclusion:**

Intraoperative communication during single‐incision laparoscopic percutaneous extraperitoneal closure demonstrates distinct phase‐specific patterns, with heightened directive teaching during the laparoscopic manipulation phase but infrequent reflective debriefing. These findings suggest that systematically incorporating debriefing into the wound closure phase could foster reflective learning, complement real‐time coaching, and enhance the overall educational impact of intraoperative experiences.

## 1 Introduction

The operating room is a unique and pivotal environment in which young surgeons develop technical skills, clinical judgment, and professional autonomy during training [1, 2]. Much of this education occurs through real‐time verbal interactions with attending surgeons during live procedures [3], making intraoperative communications a vital conduit for surgical learning and a direct influence on technical performance. In Japan, however, declining birth rates have led to a significant reduction in pediatric surgical case volumes [4], while nationwide regulations on physicians' working hours have further restricted operative exposure for trainees [5, 6]. Collectively, these factors have substantially reduced training opportunities in pediatric surgery, rendering each operative case an exceptionally valuable educational resource and highlighting the importance of maximizing the effectiveness of intraoperative teaching.

Evidence from adult surgical specialties underscores the challenges of optimizing intraoperative education. Scallon et al. reported that clinically relevant teaching occurred in fewer than half of observed operating room cases, with substantial periods devoid of meaningful educational interaction [7]. Similarly, residents frequently report limited feedback on their performance [8]. Blom et al. further noted that intraoperative verbal communication often centers narrowly on immediate, procedure‐specific guidance [9]. In contrast, comprehensive evaluations of intraoperative teaching practices in pediatric surgery remain limited. Moreover, even within adult surgery, most studies have focused primarily on guidance directed at the operating surgeon, offering little insight into how attending surgeons engage other learners, such as junior residents or medical students present in the operating room.

This study examined intraoperative verbal communication during single‐incision laparoscopic percutaneous extraperitoneal closure (SILPEC), a representative pediatric surgical procedure. By analyzing the intensity, type, and content of communication across surgical phases, we aimed to identify both strengths and opportunities for improvement in current teaching practices. As an exploratory and hypothesis‐generating study, this work seeks to provide foundational insights and generate hypotheses for future validation studies. Given the constraints on pediatric surgical training, these findings can support the development of phase‐specific, structured teaching strategies to enhance the quality and efficiency of intraoperative learning.

## 2 Materials and Methods

### 2.1 Patients

This retrospective observational study included patients aged < 16 years who underwent SILPEC at the Department of Pediatric Surgery, University of Tsukuba Hospital, Japan, between December 2024 and June 2025. Only cases with complete intraoperative video and audio recordings were analyzed. To ensure consistent evaluation across the full surgical procedure, only unilateral cases were included. Bilateral repairs, including those for asymptomatic contralateral patent processus vaginalis, were excluded to maintain comparability. Surgeries performed by surgeons beyond their 12th postgraduate year (PGY) were also excluded, as surgeons at our institution typically serve as operating surgeons until PGY 12, after which they transition into attending roles [10].

Patient characteristics reviewed included age, body weight, laterality of surgery, and history of hernia incarceration. For each case, we also recorded the PGY of the operating surgeon and operative time.

### 2.2 Intraoperative Audio Recording

In our department, intraoperative audio during SILPEC procedure is routinely recorded using a portable voice recorder (ICD‐UX570F; Sony Corporation, Tokyo, Japan) placed in the chest pocket of the operating surgeon. This practice supports postoperative video review by trainees. During the study period, all surgical team members were aware of this routine audio recording; however, they were not informed of the specific details of this research, which was conducted under an opt‐out consent process.

### 2.3 Surgical Procedure and Phases

The SILPEC procedure has been described in detail in our previous work [11]. In this study, the operation was divided into three surgical phases: umbilical incision and port placement, laparoscopic manipulation, and wound closure (Table 1). Analyses were conducted for each phase. At our institution, the attending surgeon typically acts as the scopist during Phase 2, while a junior resident (PGY 1–2) or a medical student generally participates as the second assistant.

**TABLE 1:** Definition of surgical phases for single‐incision laparoscopic percutaneous extraperitoneal closure.

Surgical phase		Start (S) and endpoint (E)
Phase 1.	Umbilical incision and port placement	S: Skin incision at the umbilicus
		E: Insertion of curved forceps into the abdominal cavity
Phase 2.	Laparoscopic manipulation	E: Removal of the port
Phase 3.	Wound closure	E: Completion of wound closure

### 2.4 Coding of Verbal Communications

For each eligible case, the extracted audio encompassed the entire surgical procedure, from skin incision to completion of wound closure. The analysis focused on the statements made by the operating surgeon and the attending surgeon. All audio recordings were reviewed and transcribed verbatim by a single pediatric surgeon (M.M.). Statements were segmented into discrete, meaningful units based on pauses or silences in the speakers' discourse. Each statement was documented along with the intended recipient. Transcribed statements were coded according to the classification framework proposed by Blom et al. [7], with modifications to reflect the context of this study. Specifically, an additional category of “Advising” was incorporated into the statement types. For content categories, “direction” and “location” were consolidated into “instrument handling,” as these categories frequently overlapped and could not be clearly distinguished. This resulted in the development of an original coding scheme (Table 2).

**TABLE 2:** Framework for categorizing intraoperative verbal statements by type and content.

Content/type	Explaining	Commanding	Advising	Questioning	Miscellaneous
Operation method	○	N/A	N/A	○	N/A
Anatomy	○	N/A	N/A	○	N/A
Instrument handling	○	○	○	○	○
Visualization	○	○	○	○	○
General	○	○	○	○	○
Private	N/A	N/A	N/A	N/A	○
Undefinable	N/A	N/A	N/A	○	○

Abbreviations: N/A: not applicable; ○: applicable.

To validate the coding system, one case not included in the study sample was first transcribed, and two pediatric surgeons (M.M. and Y.S.) jointly coded the statements using the modified scheme. After familiarization with the coding procedure, approximately 20% of the included cases were independently coded by both surgeons. Inter‐rater reliability for classification of statement type and content was substantial, with Cohen's kappa coefficients of 0.76 (95% confidence interval [CI], 0.73–0.79) and 0.67 (95% CI, 0.63–0.70), respectively. The remaining coding was performed by a single surgeon (M.M.).

### 2.5 Outcome Measures

The primary outcomes were as follows:

1. The total number of verbal statements recorded during the entire surgical procedure and within each surgical phase, as well as their distribution by content.
2. The number of verbal communications per minute from attending surgeons to operating surgeons in each surgical phase.
3. The number and distribution—by statement type and content—of verbal interactions from attending surgeons directed toward operating surgeons.

The secondary outcomes were as follows:

1. Intraoperative debriefing, defined as explanatory or advisory statements by attending surgeons during the wound closure phase (Phase 3) that reflect on the preceding laparoscopic procedure (Phase 2).
2. The number and distribution—by statement type and content—of verbal interactions from attending surgeons directed toward junior residents or medical students.

### 2.6 Statistical Analyses

Continuous variables were summarized as medians with interquartile ranges (IQRs). Categorical variables were presented as counts and percentages, or as percentages alone, depending on the context. Statistical analyses and figure generation were performed using GraphPad Prism (version 10.4.0; GraphPad Inc., La Jolla, CA, USA). Comparisons of the number of verbal communications per minute from attending surgeons to operating surgeons across surgical phases were conducted using the Friedman test, followed by pairwise Wilcoxon signed‐rank tests with Holm's adjustment for multiple comparisons.

### 2.7 Ethics

This retrospective study was conducted following the principles of the Declaration of Helsinki (2024 revision). The study protocol was approved by the Institutional Review Board of the University of Tsukuba Hospital (approval number R07‐042). Written informed consent for treatment and the use of clinical data, including intraoperative video recordings, was obtained from all patients and their guardians.

Regarding the audio recordings of intraoperative conversations among medical staff—originally collected for educational purposes—verbal explanations were provided to all personnel, and an opt‐out procedure was implemented to allow their secondary use for research. The Institutional Review Board waived the requirement for individual written informed consent for this aspect.

## 3 Results

### 3.1 Patient Characteristics

A total of 41 SILPEC procedures were performed during the study period, including 17 bilateral repairs. After excluding bilateral cases, four unilateral cases without complete audio recordings, and one unilateral case performed by a surgeon with > 12 years of postgraduate experience, 19 unilateral cases were included in the final analysis. The median (IQR) age of the patients was 24 (13.5–71) months, and the median (IQR) body weight was 11.8 (9.3–18.2) kg. Of the 19 patients, 11 (57.9%) were male, and three (15.8%) had a history of hernia incarceration. The median (IQR) PGY of the operating surgeons was 8 (7–9) years. The median (IQR) operative time, representing the duration across all three phases, was 43 (36–51.5) min. The median (IQR) duration of each surgical phase was 10.8 (8.8–14.2) min for Phase 1, 17.6 (15.1–23.1) min for Phase 2, and 12.9 (10.1–17.6) min for Phase 3 (Table 3).

**TABLE 3:** Patient characteristics and surgical outcomes.

	*n* = 19
Male, *n* (%)	11 (57.9)
Age (months), median (IQR)	24 (13.5–71)
Body weight (kg), median (IQR)	11.8 (9.3–18.2)
Left side, *n* (%)	8 (42.1)
History of incarceration, *n* (%)	3 (15.8)
Operating surgeon's PGY, median (IQR)	8 (7–9)
Operative time (min), median (IQR)	43 (36–51.5)
Phase 1	10.8 (8.8–14.2)
Phase 2	17.6 (15.1–23.1)
Phase 3	12.9 (10.1–17.6)

Abbreviations: IQR: interquartile range; PGY: postgraduate year.

### 3.2 Distribution of Intraoperative Verbal Communication

**FIGURE 1:**
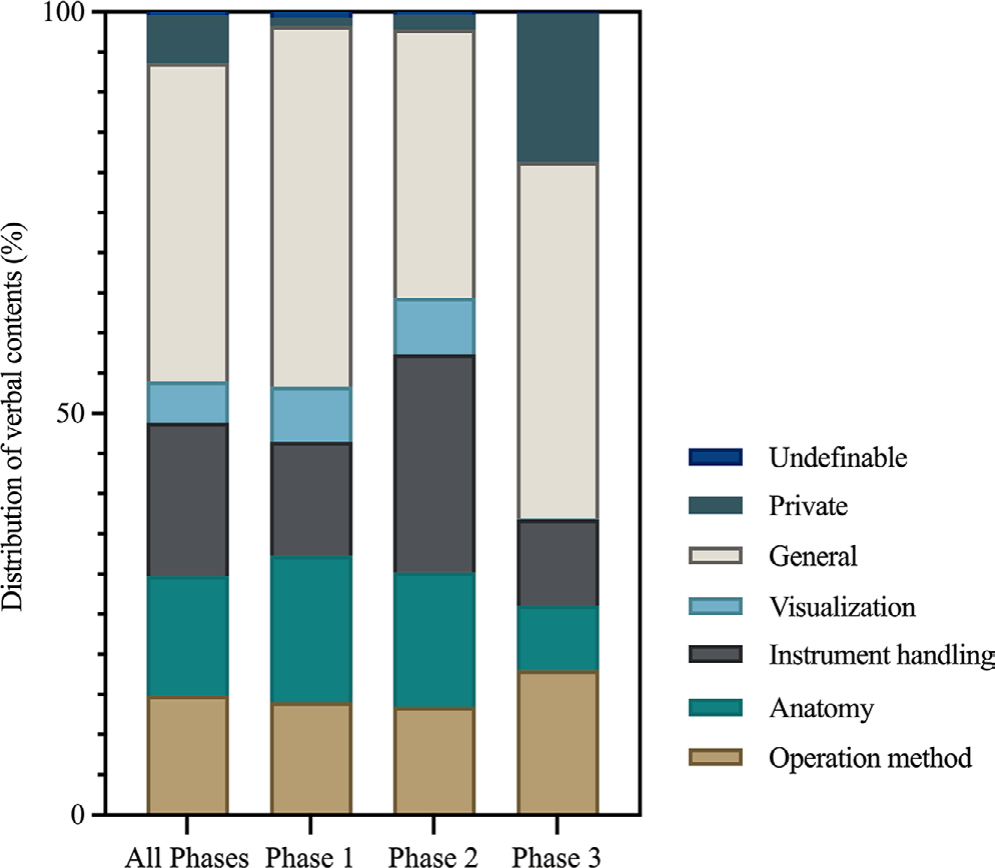
Distribution of verbal content categories across surgical phases.

Transcription of the intraoperative audio recordings yielded 7374 statements, with a median (IQR) of 346 (293–428.5) statements per case. By phase, the median (IQR) number of statements was 92 (80.5–142) in Phase 1, 149 (124–197) in Phase 2, and 86 (77–115.5) in Phase 3 (total: 2118, 3323, and 1933 statements, respectively). The overall distribution of verbal communication contents is presented in Figure 1. Across all phases, the most common category was General, accounting for 39.6% of statements, followed by Instrument handling (19.1%), Anatomy (14.9%), and Operation method (14.8%). Phase‐specific patterns were observed: in Phase 2, the proportion of statements related to Instrument handling increased to 27.1%; in Phase 3, the proportion of Private conversations rose markedly to 18.5%, while that of technical content such as Anatomy and Instrument handling decreased; and across all phases, Undefinable statements accounted for only 0.4%.

Regarding the distribution of speakers, attending surgeons accounted for 53.5% of all statements, while operating surgeons accounted for 46.5%. Phase‐specific proportions were similar: Phase 1: 53.2% (attending) vs. 46.8% (operating); Phase 2: 55.6% vs. 44.4%; and Phase 3: 50.2% vs. 49.8%.

### 3.3 Detailed Analysis of Attending Surgeons' Verbal Interactions With Operating Surgeons

Attending surgeons made 3945 verbal statements. Of these, 2858 (72.5%) were directed to operating surgeons, 557 (14.1%) to scrub nurses, 442 (11.2%) to junior residents or medical students, and 88 (2.2%) to others or to recipients that could not be defined.

**FIGURE 2:**
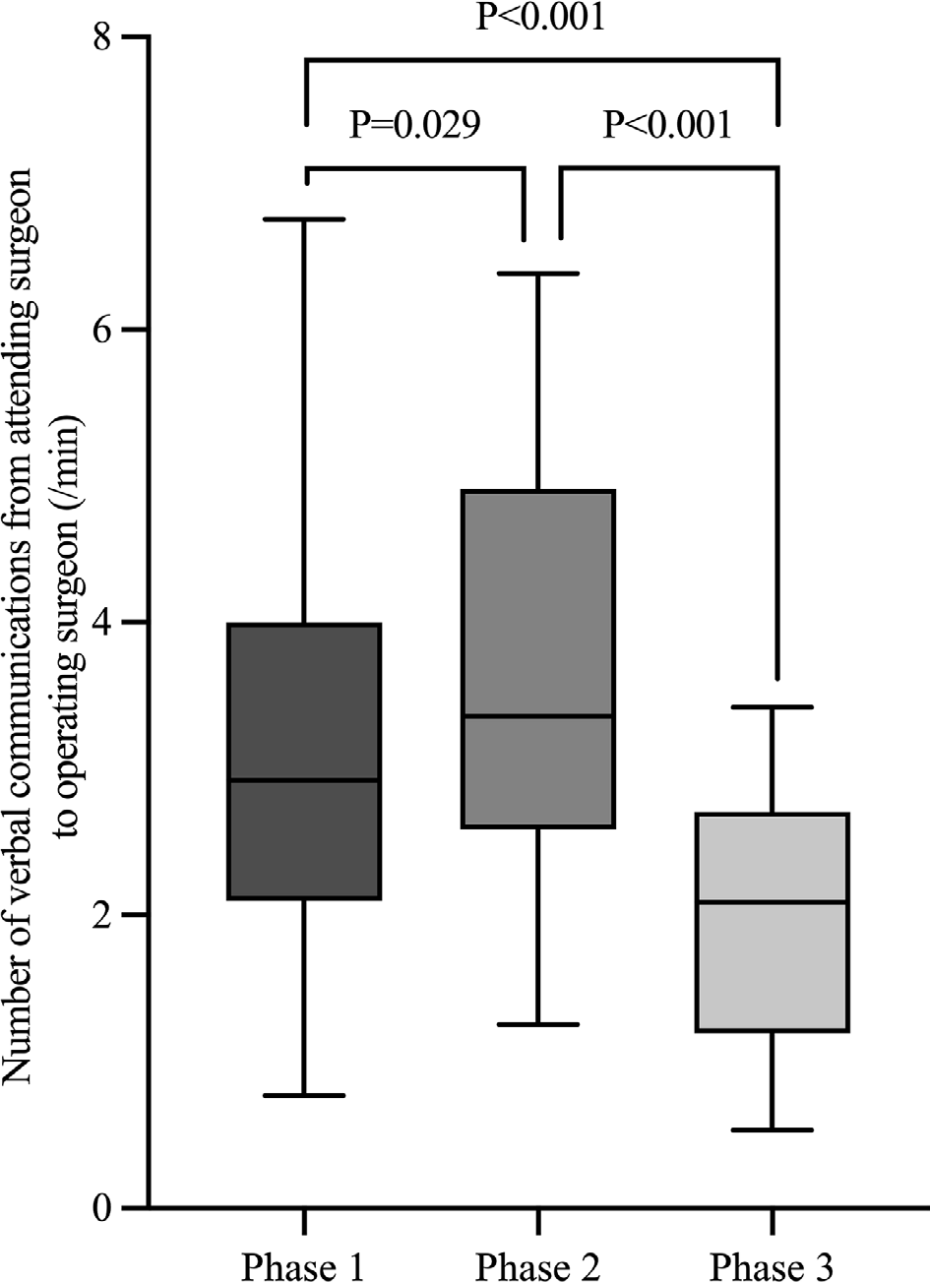
Comparison of communication intensity from attending surgeons to operating surgeons across surgical phases. Communication was most frequent during the laparoscopic manipulation phase (Phase 2), indicating heightened real‐time teaching activity compared with other phases.

Among the 2858 statements directed to operating surgeons, the median (IQR) number per case was 119 (97–167.5). The median (IQR) number of verbal communications per minute from attending surgeons to operating surgeons was 2.9 (2.2–4.0) in Phase 1, 3.4 (2.6–4.7) in Phase 2, and 2.1 (1.3–2.6) in Phase 3. Differences among phases were significant (Friedman test, *p* < 0.001). Post hoc Wilcoxon signed‐rank test with Holm's adjustment demonstrated significant differences between Phase 1 and Phase 2 (*p* = 0.029), Phase 2 and Phase 3 (*p* < 0.001), and Phase 1 and Phase 3 (*p* < 0.001) (Figure 2).

The most frequent communication type was Explaining (48.5%), followed by Miscellaneous (18.5%), Advising (12.4%), Questioning (11.1%), and Commanding (9.5%). Explaining predominated in all phases, ranging from 39.8% to 57.6% depending on the phase. Across all phases, Anatomy and Operation method were the most frequent contents within the Explaining category. Phase‐specific analysis revealed that in Phase 2 (laparoscopic manipulation), the proportion of Instrument handling within the Explaining category increased. Additionally, the proportions of Commanding (11.8%) and Advising (14.9%) rose in Phase 2 compared with those in the other phases (Table 4).

**TABLE 4:** Distribution of attending surgeons' verbal interactions to operating surgeons by type and content.

Type	Content	*n* = 2858	*n* = 743	*n* = 1512	*n* = 603
		All phases (%)	Phase 1 (%)	Phase 2 (%)	Phase 3 (%)
Explaining	Total	48.5	57.6	47.4	39.8
	Operation method	29.5	32.9	21.1	48.8
	Anatomy	32.6	38.3	30.0	30.4
	Instrument handling	19.4	13.6	27.7	4.6
	Visualization	4.8	5.8	5.9	0
	General	13.7	9.3	15.3	16.2
Commanding		9.5	8.7	11.8	4.6
Advising		12.4	10.5	14.9	8.5
Questioning		11.1	10.0	10.1	15.1
Miscellaneous		18.5	13.2	15.8	32.0

### 3.4 Intraoperative Debriefing on Laparoscopic Manipulation (Phase 2) During the Final Phase (Phase 3)

Of the 2858 statements directed to operating surgeons, 603 occurred during Phase 3. Among these, 291 were categorized as ‘Explaining’ or ‘Advising.’ However, only five statements referenced the preceding laparoscopic procedures, thereby qualifying as intraoperative debriefing. Such debriefing was observed in only two cases (10.5%), both involving operating at PGY 3.

### 3.5 Attending Surgeons' Educational Communications to Junior Residents and Medical Students

Attending surgeons directed 442 statements to junior residents or medical students, with a median (IQR) of 20 (10.5–34.5) per case. As with communications to operating surgeons, “Explaining” was the most frequent type (45.9%), followed by “Miscellaneous” (25.3%), “Commanding” (18.6%), “Questioning” (7.2%), and “Advising” (2.9%). Within the “Explaining” category, “Anatomy”‐related content was most common (39.4%), followed by content pertaining to “Operation” method (23.2%) (Table 5).

**TABLE 5:** Distribution of attending surgeons' verbal interactions with junior residents or medical students by type and content.

Type	Content	*n* = 442	*n* = 101	*n* = 151	*n* = 190
		All phases (%)	Phase 1 (%)	Phase 2 (%)	Phase 3 (%)
Explaining	Total	45.9	46.5	64.9	30.5
	Operation method	23.2	25.5	24.5	19.0
	Anatomy	39.4	51.1	44.9	20.7
	Instrument handling	9.4	6.4	2.0	24.1
	Visualization	6.4	2.1	12.3	0
	General	21.7	14.9	16.3	36.2
Commanding		18.6	25.8	18.5	14.7
Advising		2.9	5.0	0	4.2
Questioning		7.2	5.9	8.0	7.4
Miscellaneous		25.3	16.8	8.6	43.2

## 4 Discussion

This study analyzed intraoperative verbal communication during SILPEC procedures using a phase‐specific coding framework applied to audio recordings. By categorizing statements by both type and content, we examined their distribution across surgical phases and their educational relevance. To our knowledge, this is the first study to comprehensively characterize intraoperative communication in pediatric laparoscopic surgery. Three principal findings emerged. First, the frequency of communications from attending surgeons to operating surgeons was significantly higher during the laparoscopic manipulation phase (Phase 2) than during the other phases. Notably, the proportions of “commanding” and “advising” statements increased in this phase, reflecting a shift toward directive coaching. Second, intraoperative debriefing that explicitly reflected on the preceding laparoscopic phase was rare, occurring in only 2 of 19 cases (10.5%). Instead, a substantial proportion of conversation during the wound closure phase (Phase 3) consisted of non‐educational “private” talk, suggesting missed opportunities for reflective teaching. Third, attending surgeons directed educational statements not only to operating surgeons but also to junior residents and medical students, demonstrating that intraoperative teaching extended to multiple learners present in the operating room.

During Phase 2, the laparoscopic manipulation phase, attending surgeons engaged more actively with operating surgeons, as evidenced by a higher speaking rate and increased proportions of commanding and advising statements. This finding highlights the central role of real‐time technical coaching during higher‐risk maneuvers. Similar patterns have been reported in adult surgical education. Pernar et al. demonstrated that intraoperative teaching during laparoscopic cholecystectomy concentrated heavily on technical steps, underscoring the predominance of directive guidance in technically challenging portions of the operation [12]. Blom et al. further noted that because attending surgeons cannot physically indicate structures in laparoscopic surgery, deictic verbal guidance becomes essential for directing trainees through complex tasks [9]. Collectively, these findings reinforce that teaching styles adapt dynamically to the technical demands of different operative phases. Furthermore, while simulation‐based training and skill laboratories provide valuable opportunities for early acquisition of technical skills, critical aspects such as tissue handling, anatomical recognition, and the orchestration of operative flow are often more effectively learned through direct intraoperative interaction [8]. Our results support this perspective, indicating that as technical complexity increases, attending surgeons shift toward immediate, directive coaching to balance educational benefit with operative safety.

Efforts to enhance intraoperative teaching increasingly emphasize the need for structured, intentional approaches. Roberts et al. proposed the BID (Briefing–Intraoperative teaching–Debriefing) model, designed to maximize educational yield through preoperative goal setting, focused intraoperative instruction, and concise debriefing at wound closure [13]. Despite its rationale, intraoperative debriefing was observed in only 10.5% of cases in our cohort. Several factors may explain this finding. First, much of the instructional emphasis occurred as real‐time coaching during Phase 2, where technical complexity was highest. Second, the relatively advanced training level of the operating surgeons (median [IQR] PGY 8 [7, 8, 9]) suggests that many were already proficient in SILPEC, potentially reducing the perceived need for reflective teaching. Private conversation accounted for 6.0% of intraoperative communication overall, but the percentage rose to 18.5% during Phase 3. This likely reflects psychological release following the technically demanding laparoscopic phase. Roberts et al. similarly reported that private talk comprised approximately 5% of intraoperative statements, aligning with our results [14]. Although our analysis primarily viewed private talk as a potential loss of educational opportunity, qualitative studies in the operating room setting have indicated that limited informal conversation within surgical teams can foster rapport and psychological safety, supporting open discussion of professional issues and indirectly enhancing patient safety [15]. Similarly, observational research on case‐irrelevant communication in the operating room further suggests that moderate social interaction contributes to a positive team climate and stress relief after demanding phases [16]. However, excessive case‐irrelevant talk remains concerning, as it may detract from educational focus and has been linked to increased risks such as surgical‐site infection [17]. Therefore, partially incorporating structured debriefing during wound closure may help balance focused reflective learning with the benefits of limited informal interaction, without completely eliminating such exchanges. An alternative opportunity for debriefing is the postoperative period, including video‐based review sessions [18]. However, Nathwani et al. reported that US general surgery residents received postoperative feedback in only 25%–36% of cases [19]. Although both residents and attendings acknowledged its educational value, time constraints (reported by 71% of residents and 88% of attendings), lack of a private setting, and competing responsibilities at the end of the case were major barriers [19]. These findings suggest that while postoperative feedback is recognized as valuable, its consistent implementation is challenging under current resource limitations.

Medical students and junior residents often gain their first authentic exposure to surgery in the operating room, where even brief instructional interactions can meaningfully influence both learning and career trajectories. Prior research has shown that, for medical clerkship students, opportunities for active participation under appropriate supervision yield greater improvements in surgical knowledge and higher satisfaction compared with observation alone [20]. Importantly, the quality of intraoperative teaching has been shown to shape long‐term career choices: supportive and inclusive experiences foster greater interest in surgical careers, whereas negative or dismissive encounters discourage students from pursuing surgery [21]. Furthermore, the number of cases observed or the total time spent in the operating room appears less influential than the perceived quality of the educational experience [22]. These insights highlight the importance of deliberately extending teaching opportunities to junior learners. Nonetheless, attending surgeons must prioritize patient safety and fulfill their central role in guiding the operating surgeon. Within this framework, dynamically balancing attention between the primary operator and additional learners—tailored to the technical demands and progression of the procedure—provides a pragmatic strategy to optimize both educational value and operative safety.

This study has certain limitations. First, it was conducted at a single institution with a relatively small sample size and had a retrospective design. These factors may have introduced selection bias and limited the generalizability of the findings to other institutions or surgical contexts. Furthermore, this study was exploratory, descriptive, and hypothesis‐generating in nature, aiming to qualitatively characterize intraoperative communication patterns and generate hypotheses for future validation studies, rather than to test a specific hypothesis through group comparisons. Accordingly, a priori sample size estimation was not applicable. However, although the number of included cases was relatively small, the analysis encompassed more than 7000 individual statements, which allowed for meaningful characterization of phase‐specific communication trends. Second, the analysis focused exclusively on intraoperative communication and did not evaluate debriefing outside the operative period, such as post‐surgery but before patient emergence. Third, although coding reliability was assessed by two evaluators on a subset of data and demonstrated substantial agreement (Cohen's kappa), the classification process inevitably involved subjective interpretation, which may have influenced results. In addition, all of the coding was performed by a single evaluator, which could have introduced further bias. This potential limitation could be mitigated in future studies by involving multiple independent coders or incorporating automated natural language processing approaches to enhance objectivity and reproducibility. Fourth, because video and audio recording of SILPEC procedures is routine at our institution, awareness of being recorded may have altered communication patterns. Bergström et al. reported that irrelevant conversation time during endoscopic surgery decreased from 4.2% to 1.4% when both audio and video were recorded [23], and a similar effect cannot be excluded here. Finally, this study focused exclusively on SILPEC, a relatively standardized and less complex pediatric laparoscopic procedure. Communication dynamics observed in this setting may not be fully representative of more complex surgeries with greater procedural variability. Future multicenter studies with larger and more diverse cohorts are needed to validate and extend these findings.

In conclusion, this study provides the first detailed characterization of intraoperative verbal communication during pediatric SILPEC, revealing distinct phase‐specific patterns in both intensity and the distribution of statement types and contents. The laparoscopic manipulation phase was marked by increased instructional activity, particularly commanding and advising statements, whereas reflective debriefing during the wound closure phase was rare, and a considerable proportion of the conversation consisted of private talk. These findings underscore the importance of structured, phase‐specific teaching strategies in pediatric minimally invasive surgery. Integrating systematic debriefing into intraoperative practice may represent a pragmatic means of enhancing educational effectiveness while preserving operative efficiency and patient safety. Further studies are needed to validate these findings across multiple centers and to apply this framework to more complex pediatric laparoscopic procedures, thereby enhancing its generalizability and educational relevance.

## Author Contributions

**M.M.:** conceptualization, data curation, formal analysis, investigation, methodology, validation, visualization, writing – original draft. **Y.S.:** conceptualization, investigation, methodology, validation, supervision, writing – review and editing. **K.M.:** supervision, writing – review and editing. All authors have read and approved the final manuscript and agree with its content.

## Ethics Statement

The study protocol was approved by the Institutional Review Board of the University of Tsukuba Hospital (approval number R07‐042). This study was conducted following the principles of the Declaration of Helsinki (2024 revision).

## Consent

Written informed consent for treatment and the use of clinical data, including intraoperative video recordings, was obtained from all patients and their guardians. Regarding the audio recordings of intraoperative conversations among medical staff—originally collected for educational purposes—verbal explanations were provided to all involved personnel, and an opt‐out procedure was implemented to permit their secondary use for research. The Institutional Review Board waived the requirement for individual written informed consent for this aspect.

## Conflicts of Interest

The authors declare no conflicts of interest.

## Data Availability

The data that support the findings of this study are available from the corresponding author upon reasonable request.
